# Supplementary material for: p27kip1 Modulates the Morphology and Phagocytic Activity of Microglia
Source: Int J Mol Sci. 2022 Sep 9;23(18):10432. doi: 10.3390/ijms231810432 (PMC9499407; doi:10.3390/ijms231810432)
Supplement: Supplementary file 1 [file ijms-23-10432-s001.zip › ijms-1847221-supplementary.pdf]

Supplemental information

The file includes Supplementary Figures S1 to S5.

<u>Supplemental figures and legends</u>

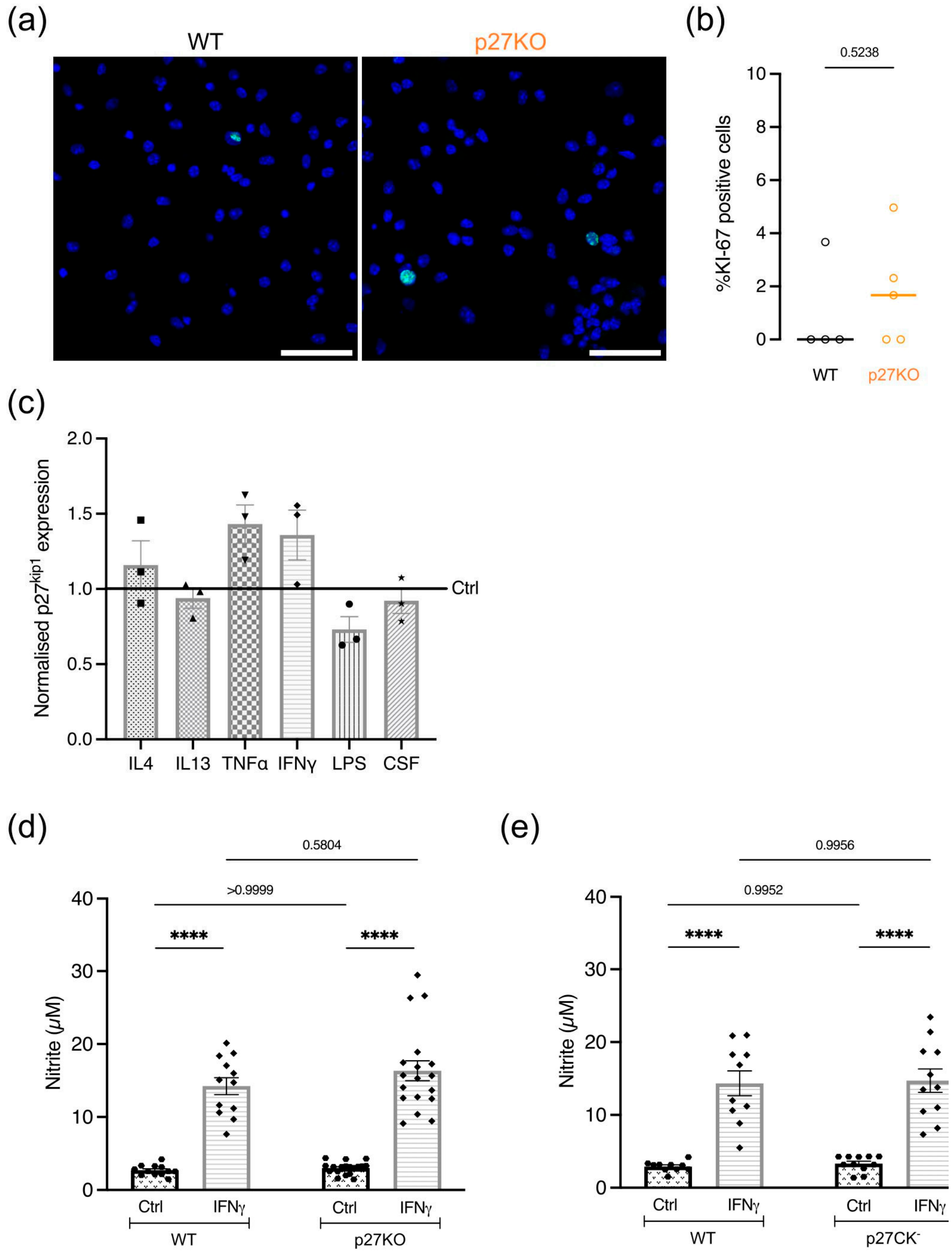

Figure S1. The constitutive absence of p27 did not affect microglial proliferation or nitrite production. (a) Representative images of forebrain isolated primary microglia nuclei (blue) showing Ki-67 expression (green). **(b)** The percentage of Ki-67 positive cells in WT (4 brains, 420 cells analyzed) and p27KO (5 brains, 673 cells analyzed) primary microglia did not significantly differ. **(c)** p27 expression in microglia was not altered upon pro- and anti-inflammatory stimulation. **(d, e)** Microglial nitrite production was measured after 24 h stimulation with IFNγ. Increased nitrite concentrations were observed but did not significantly differ between p27KO **(d)** or p27CK- **(e)** and their respective WT littermates. Sample size (d) WT: 12/7 (brains/mothers); p27KO: 18/7. Sample size (e) for

WT: 10/6; $p27CK^-$: 11/6. Horizontal bars represent the median. Data points (in c, d, e) are reported as mean ± SEM. **** p<0.0001. Multiple Mann-Whitney *U* test. Related to figure 1.

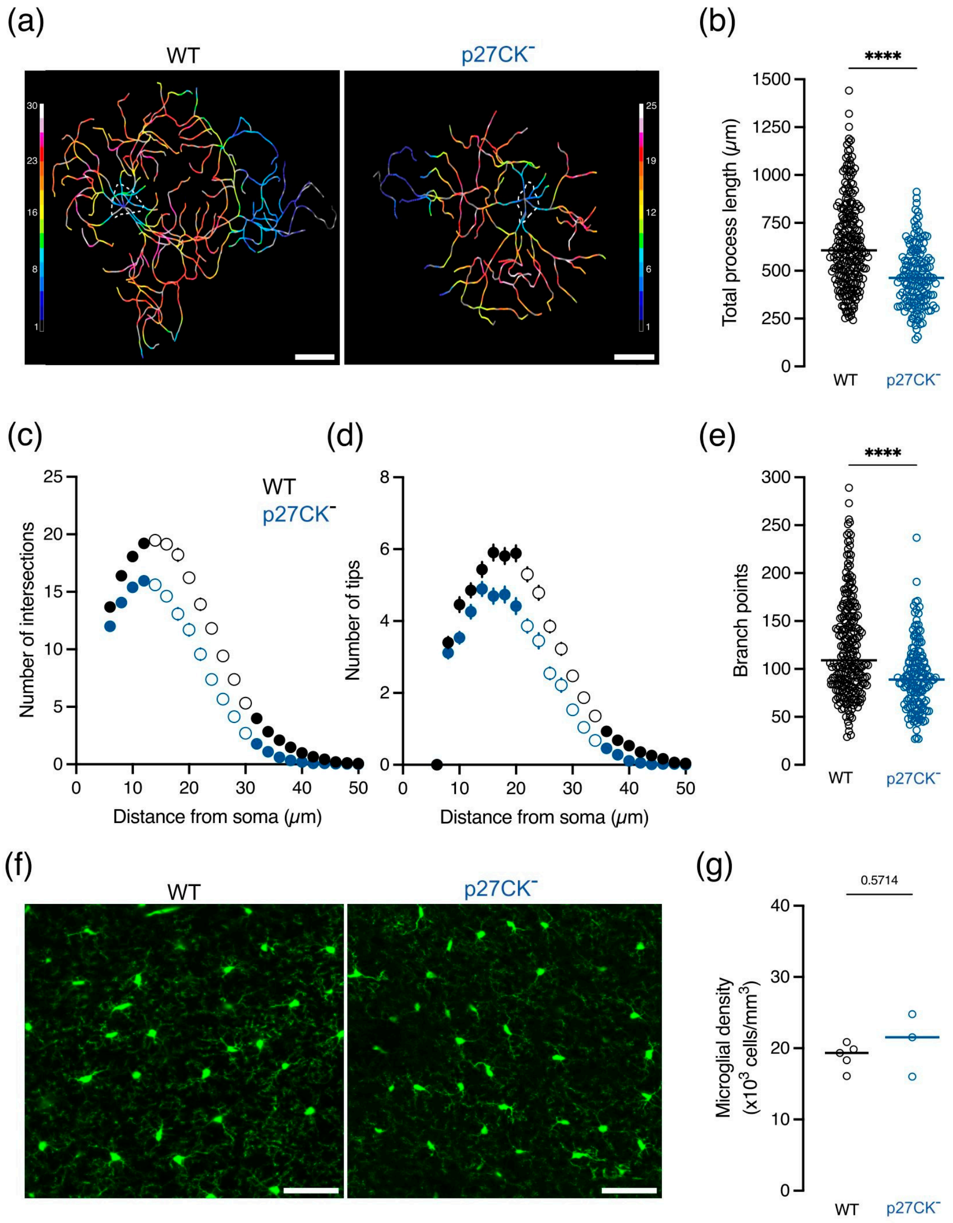

Figure S2. $p27CK^-$ microglia exhibit less ramified processes. (a–e) Morphology analysis of microglia from perfusion-fixed $CX3CR1^{eGFP/+}$p27WT (239 cells, 5 mice) and $CX3CR1^{eGFP/+}$$p27CK^-$ (155 cells, 3 mice) mice showing **(a)** Representative 3D skeletonized microglia obtained from postnatal day 21 p27WT and $p27CK^-$ microglia. Color coding represents the number of intersections (soma centered). Scale bar = 10 μm. **(b)** Total process length; **(c)** Number of process intersections within a distance from the soma; **(d)** Number of tips (terminal points) and **(e)** Branch points. **(f)** Representative images of perfusion-fixed P21 $CX3CR1^{eGFP/+}$p27WT and $CX3CR1^{eGFP/+}$$p27CK^-$ cortical microglia. Scale bar = 50 μm. **(g)** Average microglial density in the cortex of 5 p27WT and 3 $p27CK^-$ brains. ****p<0.0001. Graphs (b) and (e) represent individual microglia. Horizontal bars represent the median. Empty data points (in c, d) represent significant differences (p<0.0001). Mann-Whitney *U* test. Related to figure 2.

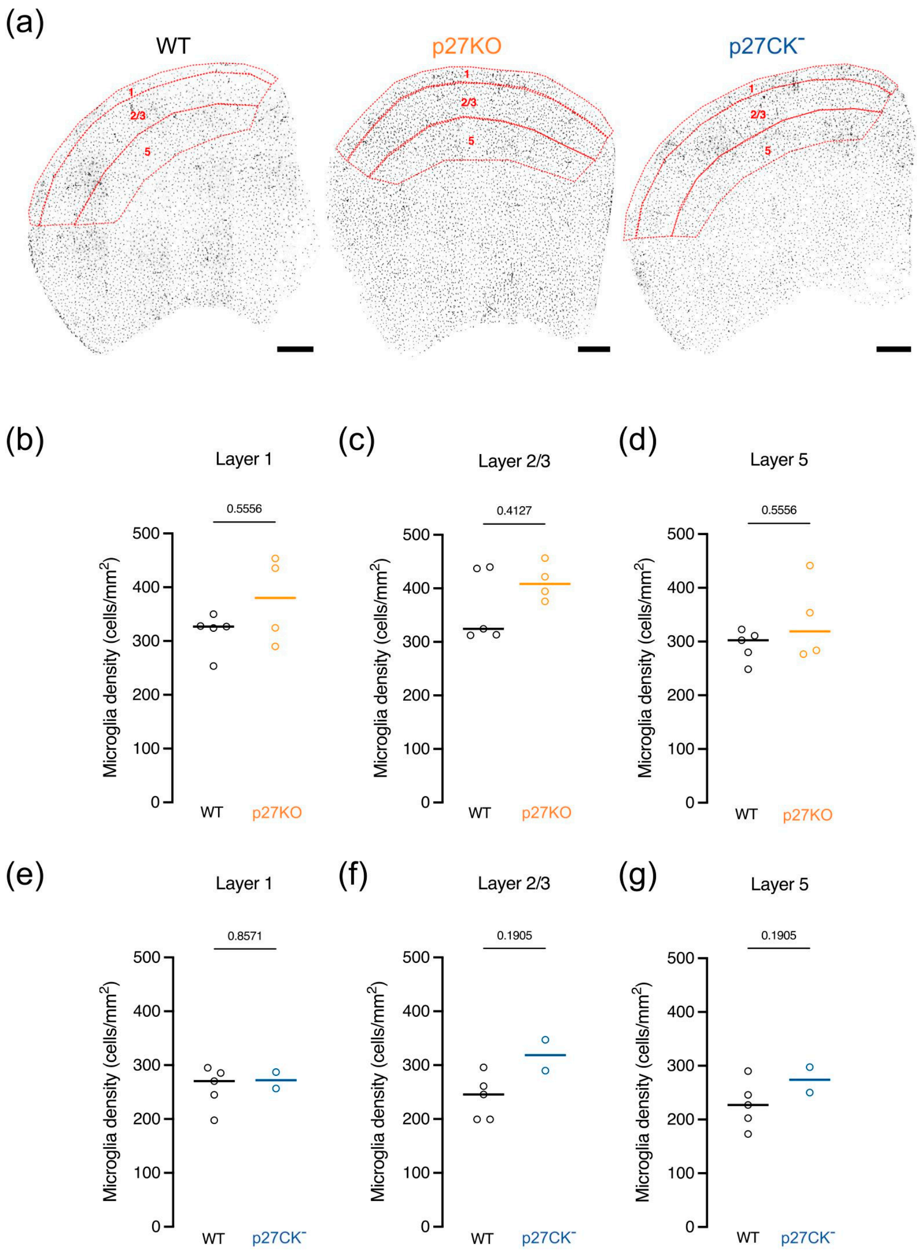

Figure S3. p27 deficiency does not impair the cortical distribution of microglia in the cerebral cortex. (a) Representative images of perfusion-fixed P21 CX3CR1$^{eGFP/+}$p27WT, CX3CR1$^{eGFP/+}$p27KO and CX3CR1$^{eGFP/+}$p27CK^{-} coronal brain slices. Density was measured in three specific regions of the secondary motor cortex, from top to bottom: layer 1, 2/3, and 5. Scale bar = 400 µm. **(b, c, d)** No difference in microglial density between p27WT (5 brains) and p27KO (4 brains) microglia in the three layers. **(e, f, g)** No difference in microglial density between p27WT (5 brains) and p27CK^{-} (3 brains) microglia in the three layers. Horizontal bars represent the median. Mann-Whitney *U* test. Related to figure 2.

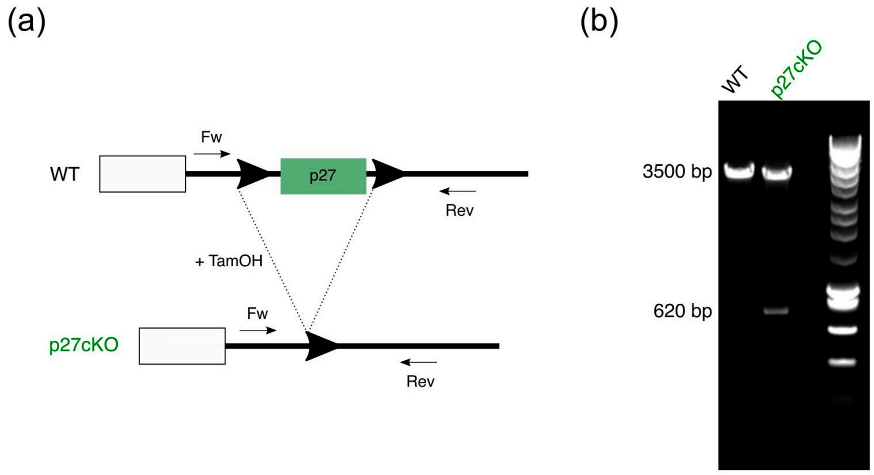

Figure S4. Generation of p27 conditional knockout. (a) Schematic representation of the p27 allele before and after recombination upon tamoxifen injection. Locations of the primers are indicated. Arrowheads represent lox sites. Fw: Forward primer, Rev: Reverse primer, TamOH: tamoxifen. **(b)** Recombination PCR. Using the primers shown in (a), the wild-type allele gives a PCR product of ~ 3500 bp representing the non-recombined allele, and the p27cKO allele shows a product of ~ 3500 bp (non-recombined allele in other cell types) and ~ 620 bp (recombined allele in microglia). Related to figure 4.

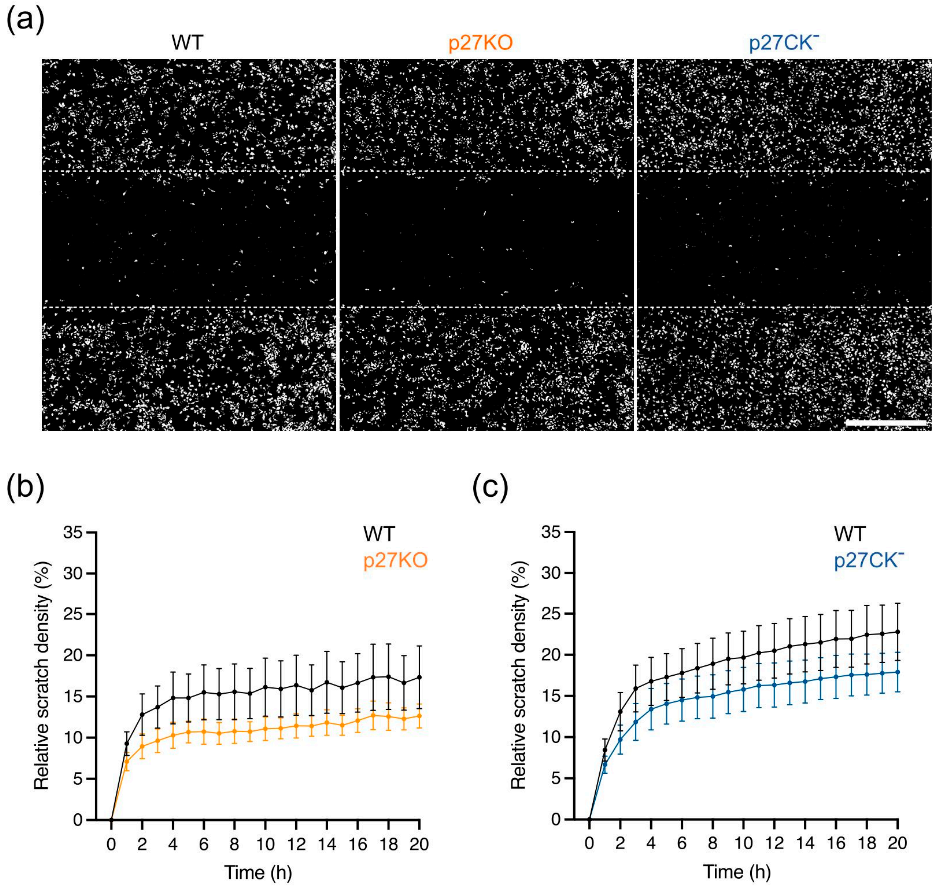

Figure S5. p27 does not control primary microglia migration *in vitro*. (a) Representative images of p27WT, p27KO, and p27CK$^-$ primary microglia after 20 h of migration towards a scratched surface. **(b, c)** Relative microglial densities in the scratched area for p27KO **(b)** and p27CK$^-$ **(c)** microglia were not significantly different compared to WT cells respectively. Sample size (b) for WT: 10/6 (brains/mothers); p27KO: 16/6. Sample size (c) for WT: 12/7; p27CK$^-$: 14/7. Data points are reported as mean ± SEM. Scale bar = 400 μm. Multiple Mann-Whitney *U* test. Related to figure 4.
